# Preventive Care and Screening Adherence Among Women Surviving Breast Cancer

**DOI:** 10.3390/cancers17172837

**Published:** 2025-08-29

**Authors:** Anthony J. Zisa, Muriel R. Statman, Marcelo M. Sleiman, Duye Liu, Adina Fleischmann, Kenneth P. Tercyak

**Affiliations:** 1 Lombardi Comprehensive Cancer Center, Georgetown University Medical Center, Washington, DC 20007, USA; 2 Sharsheret, Teaneck, NJ 07666, USA

**Keywords:** women, breast cancer, preventive services, adherence, patient navigation, community

## Abstract

**Background/Objectives**: Routine preventive care, including physical examinations, mammography, and cervical cancer screening, is critical for long-term health maintenance and recurrence surveillance among breast cancer survivors. Community-based organizations (CBOs) may play a key role in supporting adherence to these services through education, navigation, and survivorship care planning. **Methods**: A secondary analysis was conducted using data from *N* = 777 breast cancer survivors who contacted a national cancer-focused CBO for resources, including no-cost patient navigation. Preventive care adherence was measured via a composite index based on self-reported receipt of physical exams, mammograms, and Pap smears at guideline-recommended intervals. Patient-reported outcomes were assessed 30 days post-contact, including Survivorship Care Planning (SCP) receipt and quality of life (QoL). **Results**: Adherence to all three guideline-based screenings was reported by 66% of BCS, 29% adhered to two, and 6% to one or fewer. Physical exams had the highest adherence rate (97%), followed by mammograms (88%) and Pap smears (73%). Bivariate analyses showed higher adherence among younger survivors (t = 4.59, df = 711, *p* < 0.001), non-white survivors (t = −3.27, df = 267, *p* < 0.001), those in partnered relationships (t = 1.76, df = 54, *p* < 0.05), and individuals with better QoL (r = −0.09, *p* < 0.01). Receipt of SCP components was associated with a trend toward improved adherence: 56% received a care summary, 64% received follow-up instructions, and 45% received written materials (r = 0.05, *p* < 0.10). In multivariable regression adjusting for partnership status and SCP receipt, younger age (B = 1.13, *p* < 0.001), non-white race/ethnicity (B = 1.00, *p* < 0.01), and higher QoL scores (B = 0.09, *p* < 0.05) were significantly associated with greater adherence. **Conclusions**: Engagement with a CBO was associated with high levels of adherence to preventive care among breast cancer survivors—especially those who were younger, non-white, and with better QoL. SCP, particularly when delivered in written form with follow-up instructions, may support improved adherence. These findings highlight the value of CBO-led survivorship support in promoting long-term health maintenance for breast cancer survivors.

## Simple Summary

Staying healthy as a breast cancer survivor requires regular checkups and screening tests. Unfortunately, many survivors do not always receive these important follow-up services, which can lead to missed opportunities to detect problems early. This project examined women who reached out to a national community organization for support and guidance after breast cancer treatment. We wanted to learn how often they were following recommended screening practices, and what factors were linked to better follow-up care. We found that most survivors were keeping up with regular checkups, mammograms, and Pap smears, especially younger women, women from diverse backgrounds, and those reporting better overall health. Community organizations can play a key role in helping survivors stay on track with their long-term care, which may improve cancer survivorship outcomes.

## 1. Introduction

Breast cancer is the most diagnosed cancer among women in the United States, with one in eight women affected in their lifetime [1]. While incidence rates have remained relatively stable over the past several decades [1], overall survival has improved by more than 40% in the past 35 years [1,2]. As a result, there is increasing recognition of the need for comprehensive, long-term care for this expanding survivor population.

Cancer survivorship has become understood as a distinct phase of care that begins at diagnosis and extends across the lifespan [3,4]. National clinical guidelines, including those from the American Cancer Society (ACS), the American Society of Clinical Oncology (ASCO), and the National Comprehensive Cancer Network (NCCN), recommend ongoing surveillance for breast cancer survivors (BCS) through a combination of routine physical examinations, annual mammograms, and cervical cancer screening tests, based on age and individual risk profiles [5,6,7]. Surveillance is essential for early detection of recurrence and managing late effects of systemic therapy, such as cardiovascular and bone disease [6,8,9]. However, adherence to long-term surveillance guidelines remains suboptimal. Social factors, including financial constraints and insurance coverage gaps, have been linked to both diminished quality of life (QoL) and lower adherence to medical care receipt among survivors [10,11]. Additional gaps in survivorship care planning (SCP), defined as a plan provided to a patient after active treatment ends with recommendations for follow-up care, also contribute to suboptimal engagement in long-term care [12]. Fragmentation in the transition from oncology to primary care is a well-documented barrier, with survivors frequently citing a lack of clear direction for their post-treatment care [13,14,15]. These factors disproportionately affect historically underserved populations, such as racial and ethnic minorities, individuals from low-income backgrounds, and non-English speakers, contributing to disparities in outcomes and widening gaps in survivorship care that are health-harming [16,17]. Financial toxicity, defined as the problems a patient has related to the cost of medical treatment, further exacerbates these challenges by undermining access to long-term care [10,18].

Community-based organizations (CBOs) play an invaluable role in addressing these barriers for some patients, with a core component of their programs being to promote adherence to guideline-recommended preventive health practices [18]. Through targeted interventions focused on social determinants of health, CBO information and support programs have been shown to promote engagement in preventive health behaviors, including participation in routine screening, which involves not only undergoing the test itself but also navigating access [19,20]. For survivors from marginalized communities, these organizations are particularly important, as they often design programs that are no-cost and are meant to advance equitable health outcomes [19,20]. Although CBOs can make an important contribution to promoting these health practices among BCS [19,21], there remains a gap in understanding how they support adherence to cancer-specific surveillance guidelines among the population they serve. If this information were available, it could inform the design and implementation of more effective community-based survivorship interventions and reduce gaps in survivor follow-up care.

This study examined adherence to the recommended surveillance practices of physical examinations, mammograms, and Pap smears among BCS who engaged with a national cancer control CBO. It also investigated whether adherence was associated with demographic and clinical characteristics, self-reported QoL, and exposure to SCP in order to identify potential intervention targets and better understand how community-based engagement may influence follow-up. Findings from this evaluation may inform BCS interventions and guide efforts to improve their long-term care through community-based approaches.

## 2. Materials and Methods

### 2.1. Study Design

This is a secondary analysis of self-reported survey data collected over 10 years by a national CBO that offers no-cost patient navigation (PN) services to BCS at-risk for and/or surviving with breast/ovarian cancer. Data were collected 30 days following BCS’ participation in one or more of the organization’s programs, with the goal of ascertaining feedback to improve care quality and the design and delivery of health services. For the purpose of this evaluation, program participation was defined as one or more completed encounters between BCS and the CBO and/or its representatives; SCP exposure was defined as receipt of a treatment summary, follow-up instructions, or other written documentation from providers. Surveys were distributed by the CBO’s evaluation partner by email to all BCS who engaged with the CBO: data were collected using a highly secure online survey platform. Non-responders received three reminders weekly after survey initiation, and a final reminder was sent on the fourth week. After that time, and if no response was received, no further survey attempts were made. This study was reviewed and approved and deemed exempt by the Institutional Review Board at the host university due to its use of de-identified secondary data collected as part of a continuous quality improvement initiative.

### 2.2. Program Description

The CBO offers access to no-cost PN, educational materials, and BCS-focused support resources. While formal SCP, such as treatment summaries and follow-up instructions, are provided by medical providers, the CBO complements these efforts by providing education and navigation to reinforce BCS engagement. The program promotes adherence to evidence-based cancer screening and preventive care by offering tailored information, navigation to screening, and written care summaries. Services are designed to empower survivors to engage in follow-up and adopt long-term health maintenance behaviors aligned with national guidelines [22].

### 2.3. Measures

#### 2.3.1. Demographic and Clinical Information

BCS provided demographic information, including age, race, ethnicity, and partnership status. They also provided self-reported medical histories, including information on cancer previvorship (e.g., familial and/or genetic risks) and survivorship related to breast, ovarian, or other cancers. Following guidance by the Centers for Disease Control and Prevention for assessing health-related QoL, BCS reported on their overall health (1 = Excellent, 5 = Poor).

#### 2.3.2. Survivorship Care

BCS responded to a series of items assessing their SCP exposure. SCP exposure was defined as survivor-reported receipt of up to three components from their medical team: (1) a written summary of treatment received, (2) follow-up instructions for cancer surveillance, and (3) printed or written documentation of care instructions. All SCP-related items were presented in a Yes/No format and included a “Don’t know/Not sure” response option. While the CBO provides survivorship education and support, receipt of formal SCP materials largely reflects interactions between BCS and their own medical providers. From these data, a SCP exposure index score was created to unify these exposures: one point was assigned for each exposure. The ordered categorical variable had a score range from 0 to 3, with 0 indicating no exposure and 3 meaning full exposure to all of the guideline-based SCP.

To assess engagement with healthcare services, BCS were asked to identify the type of healthcare provider who currently manages the majority of their care using a drop-down list formatted with the following options: cancer surgeon, family practitioner, general surgeon, gynecologic oncologist, internist, plastic surgeon/reconstructive surgeon, medical oncologist, radiation oncologist, urologist, and “other.” Insurance coverage was assessed by asking whether BCS had health insurance that covered all or part of their cancer treatment at the time of diagnosis. Symptom burden was evaluated using two items: whether the survivor was experiencing pain related to cancer or its treatment, and whether that pain was currently under control. Pain burden was included as an indicator of ongoing symptom management needs that could impact engagement in survivorship care.

#### 2.3.3. Preventive Care

BCS were asked whether they had received a physical exam, mammogram, or Pap smear in the past two years. For each procedure, BCS used a three-option checklist to indicate whether they had not received it, received it for routine screening, or received it in response to symptoms. BCS were considered fully adherent if they reported receiving all three services for routine screening within the past two years. This two-year window reflects a conservative but inclusive application of national recommendations from the ACS, ASCO, and NCCN, which advise annual visits and age-appropriate cervical cancer screening for BCS. Next, a preventive care adherence index score was created to unify these discrete behaviors, with one point assigned for each screening received for routine purposes. The ordered categorical variable had a score range from 0 to 3, with 0 indicating no adherence and 3 reflecting behavioral adherence to all recommended preventive measures within the designated interval. This index served at the primary dependent variable of interest in statistical analyses.

### 2.4. Data Analysis Plan

Analyses were conducted in SPSS version 29.0. A priori sample size calculations were conducted to detect small to moderate effect sizes based on a projected analytic *N* > 500 and *N* < 800. Descriptive statistics were used to summarize the sample’s demographic and clinical characteristics, SCP exposure, and screening adherence and related indices. Bivariate analyses included *t*-tests and correlation coefficients to examine associations between demographics, clinical characteristics, QoL, and the SCP index with the preventive care adherence index: chi-square tests determined if the distributions of the data within the SCP and adherence indices differed than what would be expected by chance. In accordance with ACS, ASCO, and NCCN guidelines, and informed by prior literature on preventive care receipt among BCS, variables demonstrating associations with adherence at *p* < 0.10 in bivariate analyses were included in a multivariable regression model to examine factors independently associated with guideline-concordant preventive care adherence, and any secular trends were explored as well.

## 3. Results

### 3.1. Sample Characteristics

Over a ten-year period, *N* = 1983 surveys were distributed to BCS who engaged with the CBO. Of these, *N* = 1061 (53.5%) completed the 30-day post-engagement evaluation. A final analytic sample of *N* = 777 was selected based on completeness of data for the dependent variable (screening behaviors), and independent variables, including SCP exposure, QoL, and clinical features and demographics. Sample characteristics are summarized in Table 1. Among these BCS, 37% were aged ≤ 46 years, 19% identified as non-white, and 63% reported being in a partnered relationship. Nearly half (47%) carried a pathogenic *BRCA* mutation that predisposes to hereditary breast/ovarian cancer, and 22.6% rated their QoL (general health) as being fair or poor. Over half (57.5%) reported experiencing pain related to cancer or its treatment. Among those who did, 72.9% indicated that their pain was currently well-managed. Medical care was being delivered by either a primary care provider (52.3%) or an oncology specialist (47.7%).

### 3.2. Survivorship Care Planning

Based on BCS’ self-reported data, 55.9% received a written summary of their cancer treatment at the conclusion of therapy, 64.2% recalled being provided with specific follow-up instructions for their care, and 44.7% had those instructions in written form. When examined conjointly, an interesting pattern of results emerged. Specifically, nearly one-quarter of BCS did not receive any planning guidance (22.3%), and an additional one-quarter received only one piece of guidance (25.6%). The remainder of the BCS received either two (17.2%) or all three pieces of guidance (34.9%), and the resulting distribution was different than what would be expected by chance, χ^2^ (3) = 51.5, *p* < 0.001. When summed into an index score, the average number of pieces of guidance received was nearly two (*M* = 1.7, *SD* = 1.1).

### 3.3. Preventive Care Adherence

With respect to each of the three preventive care behaviors studied in this sample, physical exams were the most frequently adhered to preventive measure (99.2%), followed by mammograms (89.4%), and Pap smears (73%). Overall, 66% of BCS reported adherence to all three preventive care behaviors (physical exam, mammogram, Pap smear), 29% were adherent to two, and 6% to one or none. %). Again, the resulting distribution was different than what would be expected by chance, χ^2^ (3) = 828.9, *p* < 0.001. When summed into an index score, the average number of preventive care behaviors adhered to was between two and three (*M* = 2.5, *SD* = 0.6).

### 3.4. Bivariate Associations

At the bivariate level (see Table 1), several characteristics were associated with greater adherence to recommended preventive care. BCS who were younger (*t* = 4.38, *df* = 774, *p* < 0.001), non-white (*t* = −2.79, *df* = 771, *p* < 0.01), in a partnered relationship (*t* = 1.82, *df* = 775, *p* < 0.05), and those reporting better overall QoL (*r* = −0.09, *p* < 0.01) were more likely to be adherent. Receipt of SCP components also trended toward higher adherence (*r* = 0.05, *p* < 0.10).

### 3.5. Multivariable Regression Analysis

In a multivariable regression model adjusting for partnership status and SCP exposure, three independent variables of adherence to preventive care remained significant: BCS who were younger (ß = −0.13, *p* < 0.001), non-white (ß = 0.10, *p* < 0.01), and those reporting better QoL (ß = −0.09, *p* < 0.05) demonstrated greater adherence to guideline-based surveillance. This model, shown in Table 2, accounted for a small but significant amount of variance: Adjusted *R*^2^ = 0.04, *p* < 0.001. Importantly, no evidence of secular trends was observed.

## 4. Discussion

This study provides important insights into preventive care adherence among BCS engaged with a national CBO offering patient navigation and survivorship resources. Our findings reveal that two-thirds of participating BCS adhered to all three recommended surveillance measures (physical examination, mammography, and Pap smear) within the past two years. This rate of full adherence is notably higher than those reported in prior studies of BCS populations, which have often found suboptimal engagement in follow-up care, particularly among underserved groups [10,11,13].

Physical examination had the highest adherence, followed by mammography and Pap smears. These patterns align with prior research indicating greater uptake of breast-specific surveillance compared to cervical cancer screening [6,7]. The comparatively lower rate of Pap smear adherence suggests that even among engaged BCS populations, opportunities remain to improve the uptake of age-appropriate gynecologic care, particularly as BCS transition away from oncology-centered follow-up.

Our results also underscore the significant role of demographic and other factors in influencing adherence. Younger age, non-white race, and higher self-reported QoL were each independently associated with greater adherence to guideline-based care receipt. These findings are consistent with prior studies linking younger survivors and racial/ethnic minority groups to higher engagement in preventive behaviors when supported by targeted interventions such as those offered by CBO’s [16,21]. While underserved populations often face systemic barriers to care, our results suggest that tailored CBO navigation and educational services may help mitigate those disparities.

Exposure to SCP components, including treatment summaries and written follow-up instructions, was modestly associated with better adherence at the bivariate level. While these associations did not persist in the multivariable model, the observed trend supports earlier work showing that SCP may improve care coordination and follow-up behaviors [12,14,15]. The proportion of survivors reporting receipt of written SCP materials is encouraging, but still indicates a gap in SCP implementation that merits further attention, particularly given national guidelines recommending individualized SCP for all cancer survivors, including BCS [5,7].

These findings contribute to a growing body of literature supporting the integration of CBO-led interventions into survivorship care models. CBOs can provide low-cost, scalable, and culturally responsive programs that support BCS in navigating complex health systems and adopting long-term health maintenance behaviors. As the BCS population continues to grow, sustainable, community-centered strategies will be critical to ensuring equitable outcomes.

This study has several limitations. First, the use of self-reported data introduces the potential for recall and social desirability biases, particularly in reporting preventive care behaviors. Although some study variables had randomly missing data, the overall proportion of missingness was within a range generally considered acceptable in clinical and health services research (<5%). Second, the study relied on a convenience sample of BCS who voluntarily engaged with a national CBO, which may limit generalizability to all BCS, especially those not connected to such resources. Relatedly, the CBO’s services were provided at no-cost and this may not be externally valid (i.e., to other settings, such as fee for service). Third, the cross-sectional nature of the analysis precludes causal inference modeling regarding the direction of the relationship between SCP exposure and adherence and reverse-causality cannot be ruled out. These and other factors, including time-related variables such as months/years since breast cancer diagnosis, should be examined prospectively. Fourth, the discrete nature of the SCP and adherence indices are limited and noted, including the timing of the SCP exposures which was not ascertained: the potential for more robust assessment and alternative modeling strategies (e.g., logistic regression) may also be appropriate to further investigate relationships among the variables of interest. Fifth, while our multivariable model adjusted for key covariates that were derived from guidelines and the research literature, unmeasured confounding factors (such as differences in health literacy, provider communication, or mental health) may have influenced adherence. Finally, we did not stratify findings by time since diagnosis or treatment completion, which may affect both survivorship needs and adherence behaviors.

## 5. Conclusions

BCS who engaged with a national CBO reported high levels of adherence to preventive care recommendations, particularly when younger, non-white, or experiencing better QoL. While exposure to SCP showed a trend toward improved adherence, gaps remain in SCP implementation, especially regarding written follow-up instructions. These findings highlight the potential of CBO-led programs to support long-term BCS care and reduce disparities in cancer outcomes. Future research should explore longitudinal effects of SCP and PN support on preventive behaviors and investigate scalable strategies to integrate CBOs more systematically into survivorship care models.

## Figures and Tables

**Table 1:** Sample Characteristics and Bivariate Associations with Adherence Index (*N* = 777).

Variable	*N* (%) or *M* (*SD*)	Test Statistic
Age Band		*t* = 4.38, *df* = 774, *p* < 0.001
≤46 years	288 (37.0)	
>46 years	489 (63.0)	
Race		*t* = −2.79, *df* = 771, *p* < 0.01
White	629 (81.0)	
Non-white	148 (19.0)	
Relationship Status		*t* = 1.82, *df* = 775, *p* < 0.05
Partnered	490 (63.0)	
Not partnered	287 (37.0)	
Pathogenic *BRCA* Alteration		*t* = 0.44, *df* = 775, *p* > 0.10
Yes	365 (47.0)	
No	412 (53.0)	
General Health		*r* = −0.09, *p* < 0.01
Excellent	65 (8.4)	
Very good		
Good		
Fair	141 (18.3)	
Poor	33 (4.3)	
Pain		*t* = −0.12, *df* = 736, *p* > 0.10
Yes	447 (57.5)	
No	330 (42.5)	
Pain Under Control ^1^		
Yes	326 (72.9)	
No	121 (27.1)	
Care Provider Type		*t* = −0.05, *df* = 775, *p* > 0.10
Primary care provider	406 (52.3)	
Oncology specialist	371 (47.7)	
Survivorship Care Planning		
Written treatment summary		
Yes	434 (55.9)	
No	343 (44.1)	
Follow-up instructions		
Yes	499 (64.2)	
No	278 (35.8)	
Written follow-up instructions		
Yes	347 (44.7)	
No	430 (55.3)	
Index	1.7 (1.1)	*r* = 0.05, *p* < 0.10
Preventive Care Adherence		
Physical exam		
Yes	752 (96.2)	
No	25 (3.2)	
Mammogram		
Yes	695 (89.4)	
No	82 (10.6)	
Pap smear		
Yes	567 (73.0)	
No	210 (27.0)	
Index	2.5 (0.6)	

^1^ Among those who reported experiencing pain.

**Table 2:** Multivariable Regression of Adherence Index.

Independent Variable	Standardized Estimate (ß)	Standard Error	*t*	*p*
Age Band	−0.13	0.05	−3.45	<0.001
Race	0.10	0.06	2.65	0.008
Relationship Status	−0.06	0.05	−1.72	0.085
Quality of Life (General Health)	−0.09	0.02	−2.43	0.015
Survivorship Care Planning Index	0.02	0.02	1.12	0.26

Adjusted *R*^2^ = 0.04, *p* < 0.001.

## Data Availability

The datasets presented in this article are not readily available because they contain sensitive survey data.

## Abbreviations

The following abbreviations are used in this manuscript:

CBO	Community Based Organization
SCP	Survivorship Care Planning
BCS	Breast Cancer Survivors
QoL	Quality of Life
